# Appendicitis-Associated Pylephlebitis With Superior Mesenteric Vein Thrombosis and Portal Vein Extension: A Case Report

**DOI:** 10.7759/cureus.109618

**Published:** 2026-05-25

**Authors:** Luis Alberto Ortega-Zapata, Alejandro Hernández-Alejo, Aldo M Pacheco-Carrillo, Matías Salinas-Chapa, Diego Salinas-Rodríguez, Guillermo Elizondo-Riojas

**Affiliations:** 1 Department of Radiology, “Dr. José Eleuterio González” University Hospital, Universidad Autonoma de Nuevo Leon, Monterrey, MEX

**Keywords:** abscess, acute appendicitis, computed tomography, portal vein pylephlebitis, portomesenteric thrombosis, superior mesenteric vein thrombosis

## Abstract

Appendicitis-associated suppurative pylephlebitis with superior mesenteric vein (SMV) thrombosis is an uncommon but clinically important complication that can be identified on contrast-enhanced computed tomography. A 47-year-old man presented with seven days of right lower quadrant abdominal pain, leukocytosis, and elevated inflammatory markers. Contrast-enhanced abdominopelvic CT demonstrated acute appendicitis without CT signs of perforation or bowel ischemia, together with occlusive thrombosis of the superior mesenteric vein extending into mesenteric venous branches. The patient underwent diagnostic laparoscopy with conversion to open appendectomy for source control, followed by broad-spectrum antibiotics and anticoagulation. Follow-up CT 10 days later demonstrated persistent SMV thrombosis and a new postoperative abscess in the appendiceal bed. A subsequent CT five days later showed an interval decrease in the abscess size but progression of thrombus to the origin of the portal vein. This case highlights the importance of systematic evaluation of the portomesenteric venous system on CT in patients with appendicitis and the value of serial imaging for detecting thrombus progression and postoperative septic complications that may directly affect management and prognosis.

## Introduction

Acute appendicitis is a common cause of acute abdomen; in uncommon cases, however, it may be complicated by suppurative pylephlebitis, a septic thrombophlebitis of the portomesenteric venous system arising from an intra-abdominal infectious focus. In this setting, thrombosis most commonly involves the superior mesenteric vein (SMV), with possible extension to the portal vein. Early recognition of this complication has improved with the widespread use of contrast-enhanced computed tomography (CT), which allows simultaneous evaluation of the appendix, the portomesenteric venous system, and potential ischemic or septic complications [1].

Appendicitis-associated suppurative pylephlebitis remains diagnostically challenging because clinical manifestations may be nonspecific or disproportionate to the apparent severity of appendicitis, which can delay diagnosis. Before the widespread use of contrast-enhanced CT, diagnosis was often delayed because clinical findings were nonspecific and the condition was difficult to recognize preoperatively [2,3]. Its clinical importance lies in the risk of thrombus propagation, bowel ischemia, sepsis, and extension into the portal vein, all of which may substantially worsen outcomes if not recognized promptly [3,4].

Available evidence suggests that portomesenteric venous thrombosis is an uncommon complication of appendicitis, with an incidence of approximately 0.25% among hospitalized patients in one case series [5]. Reported treatment strategies generally combine broad-spectrum antibiotics, systemic anticoagulation, and control of the infectious source, most often by appendectomy, although management is usually individualized according to thrombus extent, septic status, and associated complications [5-8].

Because the published literature is largely limited to case reports and small series, the imaging spectrum, temporal evolution, and optimal follow-up of appendicitis-associated suppurative pylephlebitis remain incompletely characterized. We present a case of acute appendicitis complicated by SMV thrombosis within the setting of suppurative pylephlebitis, with subsequent extension to the portal vein on serial CT, highlighting the importance of systematic assessment of the portomesenteric venous system and of imaging follow-up for detecting thrombus progression and postoperative septic complications.

## Case presentation

A 47-year-old man presented to the emergency department with seven days of abdominal pain, predominantly in the right lower quadrant. On physical examination, he had right lower quadrant tenderness. Laboratory tests demonstrated leukocytosis, with a white blood cell count of 19,600/µL (reference range: 4,000-11,000/µL), neutrophilia with an absolute neutrophil count of 16,500/µL (reference range: 2,000-6,900/µL), and elevated C-reactive protein of 18.4 mg/dL (reference range: <1.0 mg/dL).

A contrast-enhanced abdominopelvic CT was performed with arterial and portal venous phase acquisitions. CT demonstrated findings consistent with acute appendicitis, including appendiceal dilatation, wall thickening, and periappendiceal inflammatory changes (Figure 1). No CT signs of perforation or bowel ischemia were identified on the initial examination.

**Figure 1:**
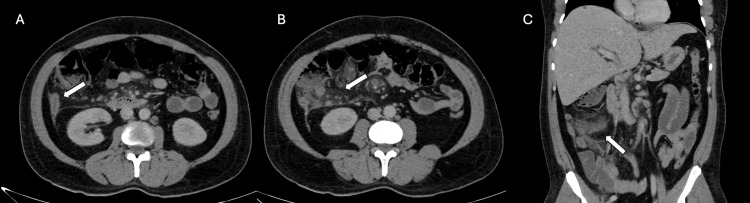
Contrast-enhanced CT in the portal venous phase demonstrating acute appendicitis. Contrast-enhanced CT in portal venous phase demonstrates a dilated appendix with mural thickening in axial (A) and coronal (C) images (arrows). Axial image (B) shows adjacent periappendiceal inflammatory fat stranding (arrow), consistent with acute appendicitis. CT, computed tomography.

The same study also demonstrated occlusive thrombosis of the SMV, with extension into mesenteric venous branches (Figure 2). At that time, there was no portal vein involvement identified on CT.

**Figure 2:**
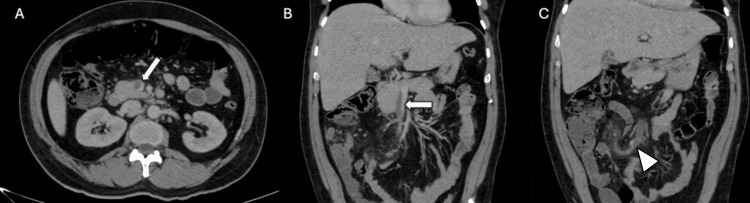
Contrast-enhanced CT demonstrating occlusive superior mesenteric vein thrombosis. Contrast-enhanced CT in portal venous phase demonstrates an intraluminal filling defect within the SMV, consistent with occlusive thrombosis, in axial (A) and coronal (B) images (arrows). Coronal image (C) demonstrates extension of the thrombus into mesenteric venous branches (arrowhead). CT, computed tomography; SMV, superior mesenteric vein.

One day after the initial CT examination, the patient underwent diagnostic laparoscopy with conversion to open appendectomy for source control. Postoperatively, broad-spectrum antibiotics and anticoagulation were initiated.

A follow-up contrast-enhanced CT obtained 10 days after surgery demonstrated persistent SMV thrombosis and a new rim-enhancing fluid collection in the appendiceal bed, consistent with a postoperative abscess (Figure 3).

**Figure 3:**
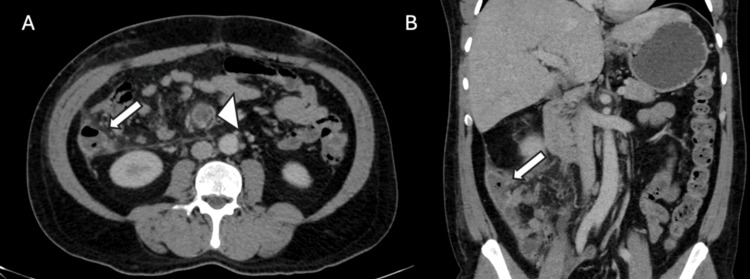
Postoperative day 10 contrast-enhanced CT demonstrating appendiceal-bed abscess and persistent superior mesenteric vein thrombosis. Follow-up contrast-enhanced CT in the portal venous phase obtained on postoperative day 10 demonstrates a rim-enhancing fluid collection in the appendiceal bed, consistent with postoperative abscess, in axial (A) and coronal (B) images (arrows). Axial image (A) also demonstrates persistent SMV thrombosis (arrowhead). CT, computed tomography; SMV, superior mesenteric vein.

A subsequent CT performed five days later showed an interval decrease in abscess size, but progression of the thrombus with extension to the origin of the portal vein (Figure 4). The patient continued with conservative management and imaging follow-up. 

**Figure 4:**
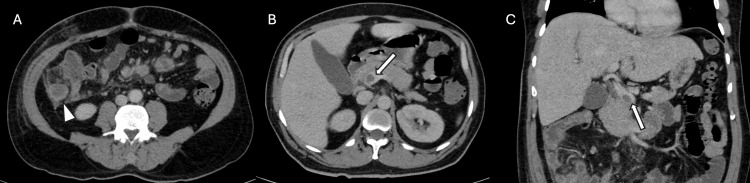
Postoperative day 15 contrast-enhanced CT demonstrating an interval decrease in appendiceal-bed abscess and thrombus progression to the portal vein. Follow-up contrast-enhanced CT in the portal venous phase obtained on postoperative day 15 demonstrates an interval decrease in the postoperative appendiceal-bed abscess on axial image (A) (arrowhead). Axial (B) and coronal (C) images demonstrate thrombus progression with extension to the origin of the portal vein (arrows). CT, computed tomography.

## Discussion

Appendicitis-associated suppurative pylephlebitis with SMV thrombosis is an uncommon but clinically important complication of acute appendicitis. Despite its rarity, this entity is relevant because delayed recognition may lead to septic progression, bowel ischemia, and extension within the portomesenteric venous system [1,3,8]. In our case, although the patient presented after seven days of symptoms, contrast-enhanced CT established the diagnosis before bowel ischemia was identified, allowing early recognition of a potentially severe complication.

The proposed mechanism is septic thrombophlebitis arising from the appendiceal infectious focus, with propagation through venous drainage into the portomesenteric venous system [1,3,8]. This explains why the imaging spectrum may range from isolated SMV thrombosis to broader involvement of the portal venous axis, particularly when infection persists or local complications develop [1,6,8]. Our case follows this pattern: the initial examination demonstrated thrombosis confined to the SMV and mesenteric venous branches, whereas follow-up CT later showed progression to the origin of the portal vein, supporting the concept that appendicitis-associated suppurative pylephlebitis may evolve over time even when portal vein involvement is absent on the index study.

Other potential causes of portomesenteric venous thrombosis include inherited or acquired hypercoagulable states, malignancy, hematologic disorders, pancreatitis, inflammatory intra-abdominal conditions, and intra-abdominal sepsis [3,5]. In the present case, the temporal association with acute appendicitis, the subsequent development of a postoperative appendiceal-bed abscess, and the pattern of thrombus progression within the setting of intra-abdominal infection supported appendicitis-associated suppurative pylephlebitis as the most likely diagnosis.

From a radiologic perspective, contrast-enhanced CT is central because it can confirm appendicitis, demonstrate venous thrombosis as an intraluminal filling defect, define its extent, and assess for associated complications such as bowel ischemia or septic collections [1,5,6]. Several reports emphasize that symptoms may be nonspecific or disproportionate to the apparent severity of appendicitis, which increases the value of systematically reviewing the portomesenteric venous system when interpreting CT in this setting [3,6,7]. In our patient, CT not only established the diagnosis of acute appendicitis but also identified occlusive SMV thrombosis on the initial examination and excluded CT findings of bowel ischemia, thereby providing information with direct therapeutic and prognostic implications.

Management described in the literature generally combines broad-spectrum antibiotics, systemic anticoagulation, and control of the infectious source, although the exact strategy is individualized according to thrombus extent, septic status, and the presence of complications [5,6,8]. Appendectomy is frequently performed for source control, while anticoagulation is commonly continued for a provoked thrombotic event, although duration varies across reports and no single standardized regimen has been established [5,6]. Our case was managed in a comparable manner, with appendectomy, antibiotics, and anticoagulation after the initial CT diagnosis. However, the subsequent development of a postoperative appendiceal-bed abscess and interval thrombus progression despite treatment highlights that clinical management does not eliminate the need for continued imaging surveillance.

An important teaching point of this case is the value of serial CT follow-up in appendicitis-associated suppurative pylephlebitis. Published reports show that follow-up imaging is useful for documenting thrombus persistence or progression, evaluating recanalization, and detecting complications that may modify management, including abscess formation and extension into the portal venous system [3,6,8]. In our patient, follow-up CT performed 10 days after surgery demonstrated persistent SMV thrombosis and a new postoperative abscess, while a subsequent study five days later documented an interval decrease in abscess size but progression of thrombus to the origin of the portal vein. These findings reinforce that radiologists should not only detect the initial thrombus but also clearly report its extent and actively assess for interval complications on follow-up studies.

Overall, this case supports a practical radiologic message: when CT demonstrates acute appendicitis, especially in patients with prolonged symptoms or a complicated clinical course, the portomesenteric venous system should be assessed systematically for suppurative pylephlebitis and SMV thrombosis. Early diagnosis, precise description of thrombus burden and extension, and recognition of postoperative septic complications may directly influence multidisciplinary treatment decisions, follow-up strategy, and prognosis.

## Conclusions

Appendicitis-associated suppurative pylephlebitis presenting with SMV thrombosis is an uncommon but clinically significant complication that may occur even in the absence of CT signs of perforation. Contrast-enhanced CT is essential not only for confirming acute appendicitis, but also for detecting venous thrombosis, defining thrombus extent, and evaluating complications such as bowel ischemia, septic collections, and progression to the portal vein.

In our case, serial CT follow-up was crucial for documenting persistent SMV thrombosis, development of a postoperative appendiceal-bed abscess, and subsequent thrombus progression to the origin of the portal vein. This case emphasizes the importance of systematic assessment of the portomesenteric venous system in CT examinations performed for suspected or confirmed appendicitis, as early recognition and accurate reporting of thrombus extent and associated septic complications may directly affect treatment, follow-up, and prognosis.
